# Gender differences in psychological morbidity and treatment in intensive care survivors - a cohort study

**DOI:** 10.1186/cc11338

**Published:** 2012-05-14

**Authors:** Anna Schandl, Matteo Bottai, Elisabeth Hellgren, Örjan Sundin, Peter Sackey

**Affiliations:** 1 Department of Anesthesiology, Surgical Services and Intensive Care Medicine, Karolinska University Hospital Solna, Sweden and The Institution of Physiology and Pharmacology, Section for Anaesthesiology and Intensive Care Medicine, Karolinska Institute, Solna, Sweden; 2 The Unit of Biostatistics, The Institution of Environmental Medicine, Karolinska Institute, Solna, Sweden; 3 Department of Psychology, Division of Social Sciences, Mid Sweden University, Östersund, Sweden

**Keywords:** post-traumatic stress, anxiety, depression, critical illness, multidisciplinary, follow-up, critical care

## Abstract

**Introduction:**

Many hospitals have initiated follow-up to facilitate rehabilitation after critical illness and intensive care, although the efficacy of such an intervention is uncertain. Studies in trauma research indicate significant differences in psychological reactions to traumatic events between men and women. Our aim, in a quasi-experimental design, was to compare psychological morbidity and treatment effects between men and women enrolled in a multidisciplinary intensive care unit (ICU) follow-up programme (follow-up group) and ICU patients not offered such follow-up (control group).

**Methods:**

Men and women treated more than four days in the ICU in 2006, before ICU follow-up started, were compared with men and women treated in 2007 and 2008, when all patients with an ICU stay of more than four days were offered ICU follow-up at 3, 6 and 12 months post-ICU. Fourteen months after ICU discharge, psychological problems were measured with Impact of Event Scale (IES) for posttraumatic stress and Hospital Anxiety and Depression Scale (HADS) for anxiety and depression.

**Results:**

Women with no follow-up reported significantly higher IES scores than men. Women in the follow-up group reported significantly lower IES scores compared to women in the control group, both in crude analysis and after adjusting for significant confounders/predictors (age, ICU length of stay and previous psychological problems). Furthermore, the 75^th ^percentile for IES and HADS-Depression scores (high scores and degree of symptoms of psychological problems) in women in the follow-up group was lower than in those without follow-up (IES: -17.4 p, *P *<.01, HADS-depression: -4.9 p, *P *<.05). For men, no significant differences were found between the no follow-up and the follow-up group.

**Conclusion:**

Psychological problems after critical illness and intensive care appear to be more common in women than in men. A multidisciplinary ICU follow-up may reduce the incidence of long-term symptoms of posttraumatic stress and depression for women.

## Introduction

Many intensive care unit (ICU) survivors suffer from physical and psychological problems after critical illness and intensive care [1]. Acute or traumatic onset of life-threatening illness, together with potentially traumatic experiences in the ICU may contribute to the development of treatable psychological problems, such as posttraumatic stress [2], anxiety and depression [3]. In general, women have more than a two-fold risk of developing posttraumatic stress disorder (PTSD) compared to men (10.4% in women versus 5% in men) [4] after being exposed to similar types of trauma. Also, women tend to recover more slowly from PTSD and are four times more likely to develop long-lasting PTSD [5]. Therefore, it may be reasonable to assume that men and women handle psychological problems after traumatic events, such as critical illness, in different ways. To facilitate rehabilitation after critical illness guidelines have been issued recommending intensive care units to offer ICU survivors physical and psychological follow-up [6-8], but the efficacy of such an intervention remains uncertain [9].

The aim of the present study was to compare long-term symptoms of posttraumatic stress, anxiety and depression in men and women enrolled in a multidisciplinary ICU follow-up programme with those not offered such help.

## Materials and methods

This quasi-experimental study was performed in a 12-bed general ICU at Karolinska University Hospital in Sweden, where a multidisciplinary ICU follow-up programme was established in 2007. Approximately 900 patients with surgical or medical diagnoses receive treatment in the ICU yearly. Ethical approval of the study protocol was received from the Regional Ethical Review Board.

### Participants

**Figure 1:**
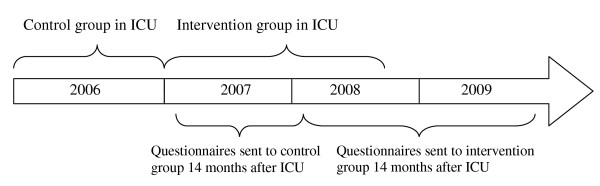
**Time points for patient enrolment**. ICU = Intensive Care Unit.

**Figure 2:**
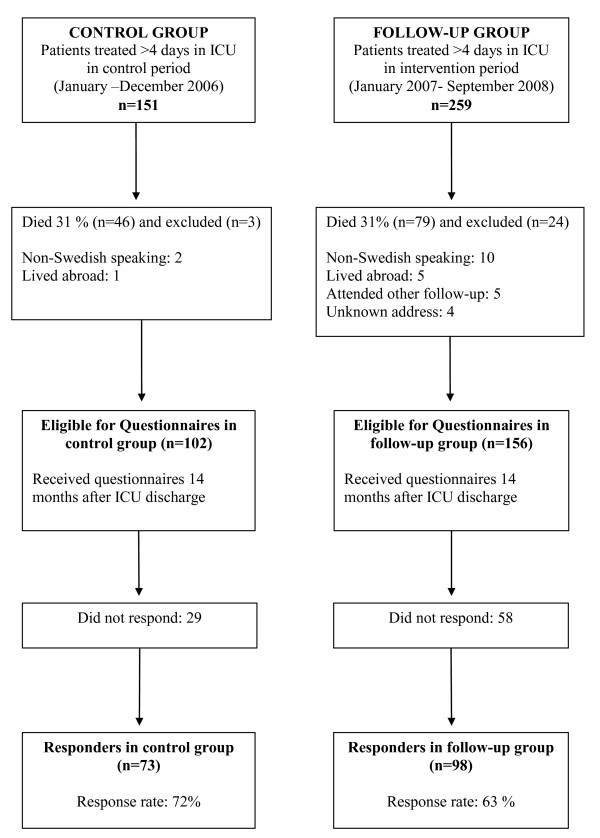
**Flow diagram of patient recruitment for control group and follow-up group**. ICU = Intensive Care Unit

Patients ≥16 years old, treated for more than 96 hours in the general ICU were consecutively enrolled in the study. Patients that did not speak Swedish and patients with no address were excluded. Patients treated from January to December 2006 (n = 151 patients) represented the control group (Figures 1 and 2). At this time, no ICU follow-up was available and patients were merely called for routine surgical or medical follow-up consultations. From January 2007 to September 2008 (n = 259 patients), all patients treated for more than four days in ICU were offered ICU follow-up. These patients represented the follow-up group. Included patients in both groups received evaluation questionnaires if still alive 14 months after ICU discharge.

### Intervention

**Figure 3:**
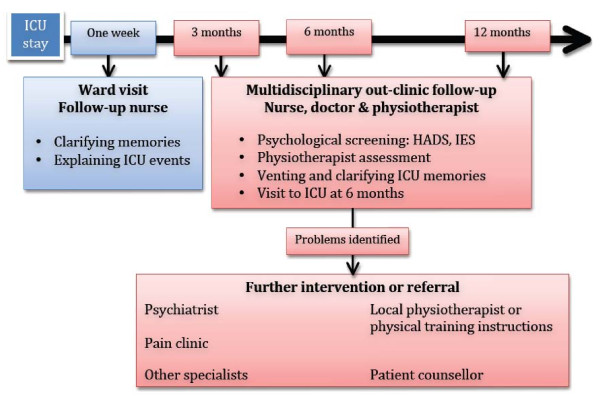
**Organization of the multidisciplinary ICU follow-up programme**. ICU, Intensive care unit; IES, Impact of Event Scale; HADS, Hospital Anxiety and Depression Scale

Patients in the follow-up group were offered ICU follow-up during the first year after intensive care. Within one week from ICU discharge, a nurse from the follow-up team visited the patients at the ward. They were briefly informed about their treatments in ICU and memories were clarified. The patients were offered multidisciplinary follow-up consultations at 3, 6 and 12 months after ICU discharge (Figure 3). Sixty-six percent of invited patients came for follow-up (18% for one visit, 48% for two or more visits) and 34% declined follow-up. At each time point patients met a nurse, a physician and a physiotherapist from the general ICU. The consultation involved recapitulating ICU-care and treatment. Memories, delusions and/or nightmares identified with the ICU-Memory-Tool [10] were discussed. At the six-month consultation patients were offered a visit of the ICU. At each time point patients were screened for psychological problems using two questionnaires: Impact of Event Scale (IES) [11] and Hospital Anxiety and Depression Scale (HADS) [12]. Patients with significant psychological problems, defined as more than 25 points in IES [11] and/or more than 10 points in any of the HADS subscales [12], were offered a referral to an appointed hospital psychiatric unit where further evaluation potentially led to treatment, for example, cognitive behavioural therapy or antidepressant therapy. Throughout the follow-up period 17 patients were referred to a psychiatrist for further psychological evaluation and treatment. Thirteen patients met the psychiatrist and four opted not to go between the time of referral and the planned appointment. Four patients were prescribed antidepressant medication, two were recommended cognitive behavioural therapy, one was referred to the family doctor for treatment and one patient received antidepressants for further follow-up by the family doctor. Ninety-three percent of patients receiving psychiatric referral came for more than one follow-up consultation, compared to 63% in non-referred patients (*P *<.05).

A physiotherapist evaluated the patient's functional status and screened for physical problems. Patients with clinically significant impairment in physical status, compared with self-reported pre-ICU physical function, were referred to a physiotherapist near the patient's home or received specific instructions for training at home. Eighty-two percent of patients receiving training instructions, referred for further physiotherapy, or both came for more than one consultation at the ICU follow-up, compared to 40% in patients with no physiotherapy intervention (*P *<.05). Further detailed information of the follow-up program is available in a previously published paper [13].

### Data collection

Demographic data and potential confounders from patients' ICU stay: Acute Physiology and Chronic Health Evaluation II (APACHE II), length of ICU-stay, comorbidity, previous psychological problems and length of sedation, were extracted from the local patient data management system and the medical charts for analysis.

### Questionnaires

The primary outcome was psychological distress in men and women 14 months after ICU discharge. Symptoms of posttraumatic stress were assessed with IES and symptoms of anxiety and depression assessed with HADS. Questionnaires were sent by postal mail to all participants in the control and follow-up groups together with an information letter about the study 14 months after individual ICU discharge in order to evaluate their psychological health (Figure 1). Written informed consent was obtained from all participants in the study. The IES measures two out of three symptom clusters of posttraumatic stress (avoidance and intrusion) associated with PTSD [11]. A total IES score above 25 indicates moderate to severe posttraumatic stress (maximum score 75). Depression and anxiety were measured with the HADS using two separate subscales (maximum subscale score 21) [12] validated in a Swedish sample [14]. Subscale scores above 10 indicate clinically significant symptoms of anxiety or depression. The ICU Memory Tool (ICU-MT) was used in order to detect possible differences in ICU memories. ICU-MT is a validated questionnaire consisting of 14 items [10] regarding the patient's memory panorama before, during and after the ICU stay. A checklist of three different memory subtypes from the ICU stay allows the patients to mark what they remember. Examples of "factual memories" are memories of family, alarms or ward rounds. "Emotional memories" include negative emotions, such as fear, pain or feelings of confusion and "delusions" are characterized by hallucinations, nightmares or dreams.

### Statistical analysis

The power analysis was based on detecting a 10-point difference in IES scores between the control group and the follow-up group: 25 p (SD 15) versus 15 p (SD 15) with >80% power at an alpha level of 0.05 [15], 100 participants were required in the follow-up group. To compensate for an estimated loss to follow-up and mortality (from ICU discharge to 14 months post ICU), 150 patients needed to be sent questionnaires in the follow-up group.

Baseline characteristics were compared with Student's *t-*test, Mann Whitney U-test and Chi^2^-test where appropriate. IES and HADS between men and women in the control and follow-up groups were compared with the Mann Whitney U-test. To assess for the hypothesized difference between men and women and to control for potential confounders (age, comorbidity, previous psychological problems, length of ICU stay, APACHE II, diagnosis groups, length of sedation) logistic quantile regression analysis was used [16]. Logistic quantile regression provides information on any specified percentile of a bounded outcome variable of interest, after adjusting for confounders. We considered the three quartiles (25th, 50th and 75th percentile) of IES and HADS. The 75^th ^percentile indicates the prevalence of more severe problems of posttraumatic stress (IES) and anxiety/depression (HADS). The follow-up intervention was included as an independent variable in all regression models. As hypothesized, gender was an important effect modifier and analyses were, therefore, performed separately for men and women. The potential confounding effect of the variables was assessed by entering variables one at a time in the models. Two variables (age and length of stay in the ICU) changed the estimated coefficient of the follow-up intervention by more than 10% and were kept in the final analysis. The presence of previous psychological problems was found not to be a confounder but was a predictor of long-term psychological problems and was, therefore, kept in the regression model. The statistical analysis was performed using Stata version 11 (StataCorp, College Station, TX, USA) and PASW Statistics18.0 (SPSS Inc, Chicago, IL, USA).

## Results

Baseline characteristics in men and women were similar in the control and follow-up groups, with no statistically significant differences in demographics (Table 1). In the control group, consisting of 151 eligible patients, 49 were lost to follow-up before 14 months. Of the remaining 102 patients, 73 responded to the questionnaires (Figure 2). In the follow-up group, 259 eligible patients spent more than four days in the ICU. A total of 103 were lost to follow-up or excluded before 14 months. Of the remaining 156 patients, 98 responded to the questionnaires (Figure 2). Questionnaire responders were similar in demographic variables compared to non-responders (data not shown).

**Table 1:** Baseline demographics of patients receiving questionnaires at 14 months post-ICU.

		Control		Follow-up	
	
		Men (n = 64)	Women (n = 38)	Men (n = 102)	Women (n = 54)
Age	Mean (SD)	52 (17)	54 (20.5)	53 (17)	52 (18)
APACHE II	Mean (SD)	21 (8)	19 (10)	23 (9)	21 (8)
Charlson Comorbidity Index*	Mean (SD)	1.4 (2.1)	1.4 (1.5)	1.2 (1.6)	1.1 (1.6)
Previous psychological problems	%	12	29	14	17
ICU-length of stay (days)	Mean (SD)	9 (7)	9 (8)	11 (7)	10 (7)
Diagnosis:					
Trauma	%	32	21	36	20
Surgical		11	11	15	19
Medical		22	26	19	13
Infection		35	42	30	48
Ventilator	%	72	79	83	81
Sedation (days)	Median (IQR)	2 (0 to 4)	2 (0 to 4)	3 (1 to 6)	3 (1 to 5)

Differences were compared with Student's *t*-test, Mann Whitney U-test and Chi^2^-test where appropriate. No statistically significant differences between control and follow-up groups, *P*-values not shown. *Comorbidity was assessed by the Charlson Comorbidity Index [23]. APACHE II, Acute Physiology and Chronic Health Evaluation II; ICU,Intensive care unit; IQR, Interquartile range p25-p75; SD, Standard deviation

Women in the control group reported higher IES scores than men in the control group (median 31 p versus 10 p, *P *<.01) (Table 2). Women invited to follow-up reported lower median IES scores and HADS-depression scores than women in the control group. As displayed in Table 3, the differences in median scores were significant at *P *<.05 for IES and HADS.

**Table 2:** Questionnaire scores in control and follow-up groups (crude data)

	Control	Follow-up	*P*-value
**Women**	**n = 27**	**n = 31**	

IES	31	20	0.01*
HADS-Anxiety	6	3	0.14
HADS-Depression	7	3	0.09
Factual memories			
Emotional memories	3	3.5	0.75
Delusional memories	1	2	0.78
	1	1	0.51

**Men**	n = 46	n = 67	

IES	10	16	0.27
HADS-Anxiety	3	4	0.78
HADS-Depression	4	4	0.47
Factual memories			
Emotional memories	2	2	0.57
Delusional memories	1	1	0.50
	0	1	0.12

Scores are presented as median values. Differences between groups were tested with Mann Whitney U-test. *Statistical significance *P *<.05

IES, Impact of Event Scale; HADS, Hospital Anxiety and Depression Scale

**Table 3:** Differences in questionnaire scores between control group and follow-up groups

	Differences between control group and follow-up group			
	
	Women		Men	
	**Crude analysis**	**Adjusted analysis**	**Crude analysis**	**Adjusted analysis**

**25^th ^percentile**				
IES	-11*	-6.6	2.0	1.9
HADS-Anxiety	0	-1.8*	-1.0	-0.5
HADS-Depression	-1.0	-1.7	0	-0.2
				
**50^th ^percentile**				
IES	-11*	-10.8*	6.1	1.8
HADS-Anxiety	-3.0	-1.2	1.0	0.4
HADS-Depression	-4.0*	-1.7	0	-0.9
				
**75^th ^percentile**				
IES	-12.1*	-17.6*	-2.0	4.4
HADS-Anxiety	-5.0	-3.2	0	-0.8
HADS-Depression	-2.8	-5.4*	-2.0	-1.0

Results presented as crude analysis and analysis adjusted for age, length of intensive care unit stay and previous psychological problems. Differences were calculated using logistic quantile regression analysis. Negative values imply lower values in the follow-up group. *Statistical significance *P *<.05

IES, Impact of Event Scale; HADS, Hospital Anxiety and Depression Scale

**Figure 4:**
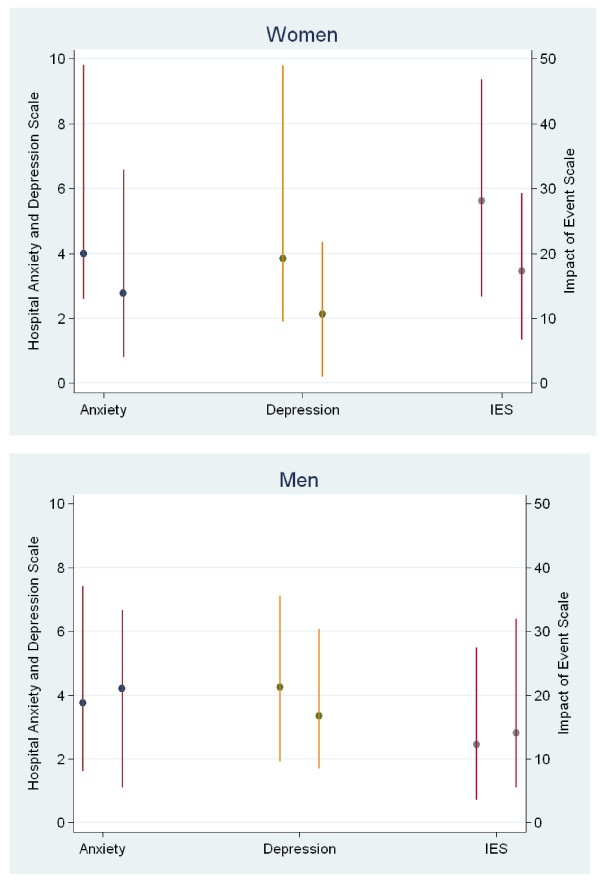
Median and interquartile range* for each outcome in controls (left-hand line) and follow-up (right-hand line). *adjusted for age, ICU- length of stay and previous psychological problems

After adjusting for age, ICU length of stay and previous psychological problems, the difference in IES between women in the control and the follow-up group remained unchanged, but the difference in median HADS-depression was smaller and no longer statistically significant (Table 3 Figure 4). For women, the 75^th ^percentile for IES and HADS-Depression scores (that is, high score/degree of symptoms of psychological problems at 14 months) was lower in the follow-up group (IES -17.6 p, *P *<.05, HADS-depression -5.4 p, *P *<.05) than in the control group (Table 3 Figure 4).

For men, no statistically significant differences were found between the control and follow-up groups (Figure 4).

Subgroup analysis of women attending psychiatrist evaluation and treatment showed a reduction of IES scores from 3 months to 14 months post-ICU in women (n = 6, median 35 p to 21 p, *P *<.05) while there was no statistically significant difference over time in men that met with a psychiatrist (n = 3, median 28 p to 10 p, *P *= 1.0).

## Discussion

In our study, women without follow-up had more psychological problems after intensive care than men, in that their IES and HADS scores were generally higher. For women offered help via a multidisciplinary follow-up service, scores were significantly lower. For men without follow-up, psychological problems were less common than in women, and posttraumatic, anxiety or depressive symptoms did not appear to be affected significantly by multidisciplinary follow-up.

Generally, experience of traumatic events in women is more strongly associated with the development of psychological problems, such as posttraumatic stress than in men [5,17]. Our findings are congruent with this observation; in the control group women reported higher IES scores than men.

One possible explanation of a reduction in PTSD-related symptoms may be the information and venting session that meeting the ICU follow-up team implied. We speculate that the information of what actually happened, and what did not happen in the ICU, together with a visit to the ICU contributed to making memories of the ICU stay less dramatic, thereby affecting posttraumatic stress development. In one sense, the follow-up resembled exposure therapy employed in the treatment of PTSD [18]; that is, the patients were exposed to the ICU environment, directly and indirectly, during less frightful circumstances. In a recently published randomized trial, recapitulating ICU care was associated with a reduction of symptoms of posttraumatic stress after critical illness [19]. ICU diaries were introduced to patients at a face-to-face meeting or sent by postal mail and discussed by phone, giving patients room to better understand their ICU stay and ask questions. During the study period, ICU diaries were written only occasionally by ICU staff and were not an integral part of the follow-up. It may be that a combination of ICU diary use and follow-up as described in our study is more efficacious than one of these strategies alone.

Another explanation of lower IES scores in women invited to follow-up may be that patients with overt symptoms were referred for psychiatric evaluation and further treatment. Women appear to be more prone to seek treatment after traumatic events [20,21] and may respond better to treatment of posttraumatic stress than men [22]. Such a gender difference in treatment efficacy in PTSD has been suggested to be due to women's ability to deal with a wider range of emotions, interpersonal relationships and a greater probability of using coping strategies [20]. Psychiatrist referral appeared to be of benefit for women in the follow-up group, although the small number of referred and treated patients with 14-month outcome data precludes firm conclusions. The psychiatrist referral was a direct consequence of high questionnaire scores noted at the follow-up consultation and had likely not been arranged without ICU follow-up and screening. Besides patients with high scores receiving a formal referral, all patients were simultaneously exposed to the follow-up, making it difficult to separate the effect of referrals from the follow-up as a whole.

As stated in the results, ICU follow-up was associated with a lower prevalence of more overt symptoms of posttraumatic stress in women 14 months after the ICU stay. Women in the follow-up group had a 17.4- point lower 75^th ^percentile IES-score compared to the control group, while the 25^th ^percentile was similar in the two groups. It is plausible that ICU patients with mild psychological problems are not as helped by ICU follow-up as those with severe problems, a possible explanation for the lack of differences in IES and HADS scores between men offered follow-up and those not offered follow-up. Measurable benefits of follow-up are likely to be most evident if interventions are performed in patients at high risk of developing problems after intensive care. UK guidelines for follow-up after intensive care [7] recommend follow-up of patients at risk of developing psychological problems. The development of early screening methods - perhaps already at ICU discharge - accurately predicting the development of later problems would be of value to improve resource allocation and to reduce late ICU-related psychological problems. Moreover, such instruments would be of value in identifying patients at risk, prior to inclusion in clinical intervention trials.

There are some limitations in this study. The use of a non-randomized, historical control group may potentially introduce bias as other treatments may have changed over time. In our study, the control data was sampled immediately prior to data for patients exposed to follow-up in order to limit the risk for bias related to changes in the ICU. We were not aware of any changes in patient admission rate, case mix, ICU staffing, ICU-length of stay or in ICU treatment routines during the study period. A regression model was used to adjust for possible differences between groups. Another limitation to consider is that this is a single centre study and results of ICU follow-up may be different in other settings. At the time of the study we followed the Swedish guidelines for ICU follow-up where first visits at two to three months were recommended. It may be that screening and treatment of psychological distress earlier than this time point would have been more efficacious. While IES is well-studied as a measure of posttraumatic stress symptoms, it is not fully diagnostic of PTSD. The cut-offs for psychiatrist evaluation and treatment in the follow-up group were decided upon with psychiatry consults but may be debated. Finally, response rates of 72% and 63% in the study groups may be considered acceptable for postal questionnaires. Despite reminder phone calls to patients, we were unable to reach a higher response rate, which is a limitation.

## Conclusions

Women surviving critical illness and intensive care appear to have more psychological problems than men and multidisciplinary ICU follow-up may reduce the incidence of long-term symptoms of posttraumatic stress and post-ICU depression for these women. Future studies to identify patients at risk are warranted, to guide clinicians and researchers to better identify patients in need for early interventions and for future clinical intervention studies.

## Key messages

• Women surviving critical illness and intensive care appear to have more psychological problems than men.

• Multidisciplinary ICU follow-up may reduce long-term symptoms of posttraumatic stress and post-ICU depression for women after critical illness.

## Abbreviations

APACHE II: Acute Physiology and Chronic Health Evaluation II; HADS: Hospital anxiety and depression scale; ICU: Intensive care unit; ICU-MT: Intensive care unit-Memory tool; IES: Impact of event scale; IQR: Interquartile range; PTSD: Posttraumatic stress disorder; SD: Standard deviation.

## Competing interests

The authors declare that they have no competing interests.

## Authors' contributions

AS, EH, ÖS and PS designed the study. AS and EH collected the data. AS, EH and PS performed the interventions together with the other members of the multidisciplinary follow-up team. AS and MB performed the statistical analysis. AS and PS wrote the draft and all other authors critically revised the manuscript and approved the final version for publication.
